# Supplementary material for: Resolving the full spectrum of human genome variation using Linked-Reads
Source: Genome Res. 2019 Apr;29(4):635–45. doi: 10.1101/gr.234443.118 (PMC6442396; doi:10.1101/gr.234443.118)
Supplement: Supplemental Material [file supp_gr.234443.118_Supplemental_longranger-2.2.zip › longranger-2.2.tar.bz2!/lr2.2-public/lib/stan/cmdstan/stan/doc/api/html/generator_8hpp.html]

Stan: src/stan/gm/generator.hpp File Reference


|  |  |
| --- | --- |
|  | Stan  2.5.0  probability, sampling & optimization |


- Main Page
- Related Pages
- Namespaces
- Classes
- Files

- File List
- File Members

All Classes Namespaces Files Functions Variables Typedefs Enumerations Enumerator Friends Macros Pages

- src
- stan
- gm

Classes |
Namespaces |
Functions

generator.hpp File Reference

`#include <boost/variant/apply_visitor.hpp>`  
`#include <boost/lexical_cast.hpp>`  
`#include <cstddef>`  
`#include <iostream>`  
`#include <ostream>`  
`#include <sstream>`  
`#include <stdexcept>`  
`#include <string>`  
`#include <vector>`  
`#include <stan/version.hpp>`  
`#include <stan/gm/ast.hpp>`

Go to the source code of this file.

|  |  |
| --- | --- |
| Classes | |
| struct | stan::gm::visgen |
|  | generic visitor with output for extension More... |
|  | |
| struct | stan::gm::expression\_visgen |
|  | |
| struct | stan::gm::printable\_visgen |
|  | |
| struct | stan::gm::var\_size\_validating\_visgen |
|  | |
| struct | stan::gm::var\_resizing\_visgen |
|  | |
| struct | stan::gm::init\_local\_var\_visgen |
|  | |
| struct | stan::gm::validate\_var\_decl\_visgen |
|  | |
| struct | stan::gm::member\_var\_decl\_visgen |
|  | |
| struct | stan::gm::local\_var\_decl\_visgen |
|  | |
| struct | stan::gm::generate\_local\_var\_init\_nan\_visgen |
|  | |
| struct | stan::gm::generate\_init\_vars\_visgen |
|  | |
| struct | stan::gm::validate\_transformed\_params\_visgen |
|  | |
| struct | stan::gm::statement\_visgen |
|  | |
| struct | stan::gm::dump\_member\_var\_visgen |
|  | |
| struct | stan::gm::set\_param\_ranges\_visgen |
|  | |
| struct | stan::gm::generate\_init\_visgen |
|  | |
| struct | stan::gm::write\_dims\_visgen |
|  | |
| struct | stan::gm::write\_param\_names\_visgen |
|  | |
| struct | stan::gm::write\_csv\_header\_visgen |
|  | |
| struct | stan::gm::constrained\_param\_names\_visgen |
|  | |
| struct | stan::gm::unconstrained\_param\_names\_visgen |
|  | |
| struct | stan::gm::write\_csv\_visgen |
|  | |
| struct | stan::gm::write\_csv\_vars\_visgen |
|  | |
| struct | stan::gm::write\_array\_visgen |
|  | |
| struct | stan::gm::write\_array\_vars\_visgen |
|  | |

|  |  |
| --- | --- |
| Namespaces | |
|  | stan |
|  | Probability, optimization and sampling library. |
|  | |
|  | stan::gm |
|  | Directed graphical model compiler. |
|  | |

|  |  |
| --- | --- |
| Functions | |
| void | stan::gm::generate\_expression (const expression &e, std::ostream &o) |
|  | |
| const std::string | stan::gm::EOL ("\n") |
|  | |
| const std::string | stan::gm::EOL2 ("\n\n") |
|  | |
| const std::string | stan::gm::INDENT (" ") |
|  | |
| const std::string | stan::gm::INDENT2 (" ") |
|  | |
| const std::string | stan::gm::INDENT3 (" ") |
|  | |
| template<typename D > | |
| bool | stan::gm::has\_lub (const D &x) |
|  | |
| template<typename D > | |
| bool | stan::gm::has\_ub (const D &x) |
|  | |
| template<typename D > | |
| bool | stan::gm::has\_lb (const D &x) |
|  | |
| template<typename T > | |
| std::string | stan::gm::to\_string (T i) |
|  | |
| void | stan::gm::generate\_indent (size\_t indent, std::ostream &o) |
|  | |
| void | stan::gm::generate\_void\_statement (const std::string &name, const size\_t indent, std::ostream &o) |
|  | |
| void | stan::gm::generate\_start\_namespace (std::string name, std::ostream &o) |
|  | |
| void | stan::gm::generate\_end\_namespace (std::ostream &o) |
|  | |
| void | stan::gm::generate\_comment (std::string const &msg, int indent, std::ostream &o) |
|  | |
| template<bool isLHS> | |
| void | stan::gm::generate\_indexed\_expr (const std::string &expr, const std::vector< expression > indexes, base\_expr\_type base\_type, size\_t e\_num\_dims, std::ostream &o) |
|  | |
| void | stan::gm::generate\_type (const std::string &base\_type, const std::vector< expression > &, size\_t end, std::ostream &o) |
|  | |
| static void | stan::gm::print\_string\_literal (std::ostream &o, const std::string &s) |
|  | |
| static void | stan::gm::print\_quoted\_expression (std::ostream &o, const expression &e) |
|  | |
| void | stan::gm::generate\_printable (const printable &p, std::ostream &o) |
|  | |
| void | stan::gm::generate\_using (const std::string &type, std::ostream &o) |
|  | |
| void | stan::gm::generate\_using\_namespace (const std::string &ns, std::ostream &o) |
|  | |
| void | stan::gm::generate\_usings (std::ostream &o) |
|  | |
| void | stan::gm::generate\_typedef (const std::string &type, const std::string &abbrev, std::ostream &o) |
|  | |
| void | stan::gm::generate\_typedefs (std::ostream &o) |
|  | |
| void | stan::gm::generate\_include (const std::string &lib\_name, std::ostream &o) |
|  | |
| void | stan::gm::generate\_includes (std::ostream &o) |
|  | |
| void | stan::gm::generate\_version\_comment (std::ostream &o) |
|  | |
| void | stan::gm::generate\_class\_decl (const std::string &model\_name, std::ostream &o) |
|  | |
| void | stan::gm::generate\_end\_class\_decl (std::ostream &o) |
|  | |
| void | stan::gm::generate\_initializer (std::ostream &o, const std::string &base\_type, const std::vector< expression > &dims, const expression &type\_arg1=expression(), const expression &type\_arg2=expression()) |
|  | |
| void | stan::gm::generate\_validate\_context\_size (std::ostream &o, const std::string &stage, const std::string &var\_name, const std::string &base\_type, const std::vector< expression > &dims, const expression &type\_arg1=expression(), const expression &type\_arg2=expression()) |
|  | |
| void | stan::gm::generate\_validate\_positive (const std::string &var\_name, const expression &expr, std::ostream &o) |
|  | |
| void | stan::gm::generate\_initialization (std::ostream &o, const std::string &var\_name, const std::string &base\_type, const std::vector< expression > &dims, const expression &type\_arg1=expression(), const expression &type\_arg2=expression()) |
|  | |
| void | stan::gm::generate\_var\_resizing (const std::vector< var\_decl > &vs, std::ostream &o) |
|  | |
| const std::vector< expression > | stan::gm::EMPTY\_EXP\_VECTOR (0) |
|  | |
| void | stan::gm::generate\_local\_var\_inits (std::vector< var\_decl > vs, bool is\_var, bool declare\_vars, std::ostream &o) |
|  | |
| void | stan::gm::generate\_public\_decl (std::ostream &o) |
|  | |
| void | stan::gm::generate\_private\_decl (std::ostream &o) |
|  | |
| void | stan::gm::generate\_validate\_var\_decl (const var\_decl &decl, int indent, std::ostream &o) |
|  | |
| void | stan::gm::generate\_validate\_var\_decls (const std::vector< var\_decl > decls, int indent, std::ostream &o) |
|  | |
| void | stan::gm::generate\_member\_var\_decls (const std::vector< var\_decl > &vs, int indent, std::ostream &o) |
|  | |
| void | stan::gm::generate\_local\_var\_decls (const std::vector< var\_decl > &vs, int indent, std::ostream &o, bool is\_var, bool is\_fun\_return) |
|  | |
| void | stan::gm::generate\_local\_var\_init\_nan (const std::vector< var\_decl > &vs, int indent, std::ostream &o, bool is\_var, bool is\_fun\_return) |
|  | |
| void | stan::gm::generate\_init\_vars (const std::vector< var\_decl > &vs, int indent, std::ostream &o) |
|  | |
| void | stan::gm::generate\_validate\_transformed\_params (const std::vector< var\_decl > &vs, int indent, std::ostream &o) |
|  | |
| void | stan::gm::generate\_statement (statement const &s, int indent, std::ostream &o, bool include\_sampling, bool is\_var, bool is\_fun\_return) |
|  | |
| void | stan::gm::generate\_statements (const std::vector< statement > &ss, int indent, std::ostream &o, bool include\_sampling, bool is\_var, bool is\_fun\_return) |
|  | |
| void | stan::gm::generate\_log\_prob (program const &p, std::ostream &o) |
|  | |
| void | stan::gm::suppress\_warning (const std::string &indent, const std::string &var\_name, std::ostream &o) |
|  | |
| void | stan::gm::generate\_member\_var\_inits (const std::vector< var\_decl > &vs, std::ostream &o) |
|  | |
| void | stan::gm::generate\_destructor (const std::string &model\_name, std::ostream &o) |
|  | |
| void | stan::gm::generate\_set\_param\_ranges (const std::vector< var\_decl > &var\_decls, std::ostream &o) |
|  | |
| void | stan::gm::generate\_constructor (const program &prog, const std::string &model\_name, std::ostream &o) |
|  | |
| void | stan::gm::generate\_init\_method (const std::vector< var\_decl > &vs, std::ostream &o) |
|  | |
| void | stan::gm::generate\_dims\_method (const program &prog, std::ostream &o) |
|  | |
| void | stan::gm::generate\_param\_names\_method (const program &prog, std::ostream &o) |
|  | |
| void | stan::gm::generate\_write\_csv\_header\_method (const program &prog, std::ostream &o) |
|  | |
| void | stan::gm::generate\_constrained\_param\_names\_method (const program &prog, std::ostream &o) |
|  | |
| void | stan::gm::generate\_unconstrained\_param\_names\_method (const program &prog, std::ostream &o) |
|  | |
| void | stan::gm::generate\_write\_csv\_method (const program &prog, const std::string &model\_name, std::ostream &o) |
|  | |
| void | stan::gm::generate\_write\_array\_method (const program &prog, const std::string &model\_name, std::ostream &o) |
|  | |
| void | stan::gm::generate\_model\_name\_method (const std::string &model\_name, std::ostream &out) |
|  | |
| void | stan::gm::generate\_model\_typedef (const std::string &model\_name, std::ostream &out) |
|  | |
| void | stan::gm::generate\_bare\_type (const expr\_type &t, const std::string &scalar\_t\_name, std::ostream &out) |
|  | |
| void | stan::gm::generate\_arg\_decl (bool gen\_const, bool gen\_ref, const arg\_decl &decl, const std::string &scalar\_t\_name, std::ostream &out) |
|  | |
| bool | stan::gm::has\_only\_int\_args (const function\_decl\_def &fun) |
|  | |
| std::string | stan::gm::fun\_scalar\_type (const function\_decl\_def &fun, bool is\_lp) |
|  | |
| bool | stan::gm::needs\_template\_params (const function\_decl\_def &fun) |
|  | |
| void | stan::gm::generate\_function\_template\_parameters (const function\_decl\_def &fun, bool is\_rng, bool is\_lp, bool is\_log, std::ostream &out) |
|  | |
| void | stan::gm::generate\_function\_inline\_return\_type (const function\_decl\_def &fun, const std::string &scalar\_t\_name, std::ostream &out) |
|  | |
| void | stan::gm::generate\_function\_name (const function\_decl\_def &fun, std::ostream &out) |
|  | |
| void | stan::gm::generate\_function\_arguments (const function\_decl\_def &fun, bool is\_rng, bool is\_lp, bool is\_log, std::ostream &out) |
|  | |
| void | stan::gm::generate\_functor\_arguments (const function\_decl\_def &fun, bool is\_rng, bool is\_lp, bool is\_log, std::ostream &out) |
|  | |
| void | stan::gm::generate\_function\_body (const function\_decl\_def &fun, const std::string &scalar\_t\_name, std::ostream &out) |
|  | |
| void | stan::gm::generate\_propto\_default\_function\_body (const function\_decl\_def &fun, std::ostream &out) |
|  | |
| void | stan::gm::generate\_propto\_default\_function (const function\_decl\_def &fun, const std::string &scalar\_t\_name, std::ostream &out) |
|  | |
| void | stan::gm::generate\_function (const function\_decl\_def &fun, std::ostream &out) |
|  | Generate the specified function and optionally its default for propto=false for functions ending in \_log. More... |
|  | |
| void | stan::gm::generate\_function\_functor (const function\_decl\_def &fun, std::ostream &out) |
|  | |
| void | stan::gm::generate\_functions (const std::vector< function\_decl\_def > &funs, std::ostream &out) |
|  | |
| void | stan::gm::generate\_cpp (const program &prog, const std::string &model\_name, std::ostream &out) |
|  | |


---

[ Stan Home Page ]

*© 2011–2014,
Stan Development Team.*
